# Antenatal care packages with reduced visits and perinatal mortality: a secondary analysis of the WHO Antenatal Care Trial

**DOI:** 10.1186/1742-4755-10-19

**Published:** 2013-04-12

**Authors:** Joshua P Vogel, Ndema Abu Habib, João Paulo Souza, A Metin Gülmezoglu, Therese Dowswell, Guillermo Carroli, Hassan S Baaqeel, Pisake Lumbiganon, Gilda Piaggio, Olufemi T Oladapo

**Affiliations:** 1 School of Population Health, Faculty of Medicine, Dentistry and Health Sciences, University of Western Australia, 35 Stirling Highway, Crawley, 6009, Australia; 2 UNDP/UNFPA/UNICEF/WHO/World Bank Special Programme of Research, Development and Research Training in Human Reproduction (HRP), Department of Reproductive Health and Research, World Health Organization, Avenue Appia 20, Geneva, CH-1211, Switzerland; 3 Department of Women’s Health, University of Liverpool, Liverpool Women’s Hospital, Crown Street, Liverpool 8, UK; 4 Centro Rosarino de Estudios Perinatales (CREP), Moreno 878, Rosario, 2000, Argentina; 5 King Saud bin Abdulaziz University for Health Sciences, College of Medicine, Jeddah, 21423, Saudi Arabia; 6 Department of Obstetrics and Gynaecology, Faculty of Medicine, Khon Kaen University, Khon Kaen, 40002, Thailand; 7 Statistika Consultoria, São Paulo, Brazil, Divonne-les-Bains, France; 8 Department of Obstetrics and Gynaecology, Olabisi Onabanjo University Teaching Hospital, Sagamu, P.M.B. 2001, Nigeria

**Keywords:** Antenatal care, Perinatal mortality, WHO, Developing country

## Abstract

**Background:**

In 2001, the WHO Antenatal Care Trial (WHOACT) concluded that an antenatal care package of evidence-based screening, therapeutic interventions and education across four antenatal visits for low-risk women was not inferior to standard antenatal care and may reduce cost. However, an updated Cochrane review in 2010 identified an increased risk of perinatal mortality of borderline statistical significance in three cluster-randomized trials (including the WHOACT) in developing countries. We conducted a secondary analysis of the WHOACT data to determine the relationship between the reduced visits, goal-oriented antenatal care package and perinatal mortality.

**Methods:**

Exploratory analyses were conducted to assess the effect of baseline risk and timing of perinatal death. Women were stratified by baseline risk to assess differences between intervention and control groups. We used linear modeling and Poisson regression to determine the relative risk of fetal death, neonatal death and perinatal mortality by gestational age.

**Results:**

12,568 women attended the 27 intervention clinics and 11,958 women attended the 26 control clinics. 6,160 women were high risk and 18,365 women were low risk. There were 161 fetal deaths (1.4%) in the intervention group compared to 119 fetal deaths in the control group (1.1%) with an increased overall adjusted relative risk of fetal death (Adjusted RR 1.27; 95% CI 1.03, 1.58). This was attributable to an increased relative risk of fetal death between 32 and 36 weeks of gestation (Adjusted RR 2.24; 95% CI 1.42, 3.53) which was statistically significant for high and low risk groups.

**Conclusion:**

It is plausible the increased risk of fetal death between 32 and 36 weeks gestation could be due to reduced number of visits, however heterogeneity in study populations or differences in quality of care and timing of visits could also be playing a role. Monitoring maternal, fetal and neonatal outcomes when implementing antenatal care protocols is essential. Implementing reduced visit antenatal care packages demands careful monitoring of maternal and perinatal outcomes, especially fetal death.

## Introduction

Many attempts have been made to rationalize the content, frequency and implementation of antenatal care in response to new evidence and technologies [1-4] and systematic reviews have examined the effectiveness of individual components of antenatal care programs [5-9]. Evidence-based, validated and safe antenatal care packages recommending fewer visits for low-risk women have large implications for developing countries, where resources are scarce and antenatal care poses both financial and logistical challenges to patients and health systems.

In 2001, Villar and colleagues published the findings of the WHO Antenatal Care Trial (hereafter referred to as the WHO ANC Trial), a multi-center, cluster-randomized trial of an evidence based, reduced visit package of antenatal care in Thailand, Cuba, Saudi Arabia and Argentina [10]. The trial found that a reduction in the number of visits and application of goal oriented, effective antenatal activities was not inferior to standard antenatal care packages in the risk of adverse outcomes for mothers and newborns [10]. The authors also stated that the widespread application of the reduced visit package might, in some settings, reduce cost.

In 2010, the Cochrane systematic review comparing the effects of reduced visits, goal-oriented antenatal care programs with the standard model of care for low-risk women was updated [4]. This review included seven trials of more than 60,000 women, four in high-income countries with individual randomization and three in low- and middle-income countries with cluster randomization, including the WHO ANC Trial. The four individually randomized trials defined reduced frequency as six to eight visits, whereas the three clusters randomized trials used a reduced schedule of four to six visits. Primary outcomes were maternal death, pre-eclampsia, perinatal death, preterm birth and small for gestational age. The reviewers found a borderline significant increase in perinatal mortality for women randomized to reduced visits compared with standard care (RR 1.14; 95% CI 1.00, 1.31). On sub-group analysis, the number of perinatal deaths in the individually randomized trials was relatively low. However, the three cluster-randomized trials consistently showed slightly higher perinatal mortality in the reduced visits group (RR 1.15; 95% CI 1.01, 1.32).

WHO convened a technical consultation in November 2010 to discuss the implications of these findings and released a statement [11]. The technical consultation was unable to identify a specific cause for the increased risk of perinatal mortality identified in the review. Possible factors discussed were differences in population, baseline risk and gaps between visits in the second and third trimester of pregnancy being too wide for timely identification of fetal ill-health and appropriate responses, or simply a chance effect. The consultation recommended that researchers from the three trials in question undertake secondary analyses of their datasets to identify possible causes. This secondary analysis of the WHO ANC Trial data explores the relationship between the reduced visits antenatal care package and perinatal mortality.

## Methods

The design of the WHO ANC Trial is described in detail elsewhere [10,12]. In brief, antenatal clinics were randomized to deliver the new antenatal care package or the standard antenatal care protocol offered in accordance with local guidelines. The new antenatal package combined evidence-based screening, therapeutic interventions and maternal education across four antenatal visits for low-risk women. A classifying form was used by the clinics randomized to the new package to identify any maternal medical and obstetric conditions and these women received additional antenatal care appropriate to that condition. The primary fetal/neonatal outcome was low birth weight, the primary maternal outcome was a maternal morbidity index defined as the presence of at least one of the following: pre-eclampsia/eclampsia, severe postpartum anemia and treated urinary tract infections/pyelonephritis. Maternal and perinatal secondary outcomes included among others fetal and neonatal mortality. The sample size calculations for the trial were based solely on the primary outcomes, since the mortality end-points would have required a very large number of participants [10].

A total of 53 clinics in Khon Kaen (Thailand), Havana (Cuba), Jeddah (Saudi Arabia), and Rosario (Argentina) were randomized. 12,568 women attended the 27 intervention clinics for a median of five visits. 11,958 women attended the 26 standard model clinics for a median of eight visits. Rates of neonatal mortality (0.6% for the new model vs. 0.7% for the standard model) were relatively similar between groups, but the rates of overall fetal death (1.4% for the new model vs. 1.1% for the standard model) were not.

For this secondary analysis, only singleton births were included. Women were stratified by baseline maternal risk to determine if differences in maternal characteristics between the intervention and control groups contributed to the risk of perinatal mortality. Fetal death was stratified by gestational age and compared between intervention and control groups to explore differences in risks according to timing of fetal death. We used generalized linear modeling and robust variance Poisson regression modeling (adjusted for clustering within clinics) to determine relative risks for perinatal mortality and fetal death by gestational age period. All analyses were conducted using SAS software v9.2 (SAS Institute Inc., Cary, NC, USA).

## Results

In the WHO ANC Trial, 6,160 women were high risk and 18,365 were low risk. Table 1 shows selected maternal characteristics stratified by risk and by group. Low-risk women were less likely to smoke and were younger and more educated. Robust variance Poisson regression modeling was used to compare perinatal outcomes between the new and standard model groups, adjusted for maternal risk and other significant covariates from univariate analysis. Table 2 shows that adjusted relative risk of perinatal death is higher in the new model (Adjusted RR 1.18; 95% CI 1.01, 1.37). This is due to an increased relative risk of fetal death (Adjusted RR 1.27; 95% CI 1.03, 1.58), which can in turn be attributed to an increased risk of fetal death at less than 36 weeks (Adjusted RR 1.64; 95% CI 1.27, 2.11). Crude and adjusted relative risks were consistent for all outcomes.

**Table 1:** Characteristics of high- and low-risk women stratified by model of antenatal care

**Characteristics**	**High-risk women N = 6,160**		**Low-risk women N = 18,365**	
	**New model**	**Standard model**	**New model**	**Standard model**
	**N = 3,287**	**N = 2,873**	**N = 9,281**	**N = 9,084**
**Demographics**				
Married or stable union	2,981 (90.8)	2,544 (89.1)	8,628 (93.0)	8.297 (91.4)
Education less than primary	720 (21.9)	591 (20.6)	1,484 (16.0)	1,291 (14.2)
Smoking during pregnancy	493 (15.0)	460 (16.0)	815 (8.8)	1,035 (11.4)
Substance abuse	64 (2.0)	41 (1.4)	-	-
Ratio persons per room in house (mean, SD)	2.5 (1.4)	2.4 (1.3)	2.4 (1.3)	2.4 (1.2)
Maternal age in years (mean, SD)	26.8 (7.0)	26.7 (7.1)	25.1 (5.5)	24.7 (5.5)
**Outcome of previous pregnancy**				
Abortion	887 (27.0)	716 (24.9)	1,794 (19.3)	1,838 (20.2)
Fetal death	87 (2.7)	68 (2.4)	5 (0.05)	6 (0.07)
Low birth weight (<2500 g)	374 (11.4)	384 (13.4)	-	-
Neonatal death	61 (1.9)	45 (1.6)	5 (0.05)	2 (0.02)
Hospital admission for hypertension or pre-eclampsia/eclampsia	215 (6.5)	132 (4.6)	-	-
**Reproductive History**				
Any previous low birth weight baby	534 (16.3)	537 (18.7)	181 (2.0)	165 (1.8)
Any previous surgery of reproductive tract	227 (6.9)	351 (12.2)	-	-
Any previous abortion	1,443 (50.4)	1,208 (48.9)	2,594 (36.8)	2,601 (38.8)
Any previous fetal death or neonatal loss	563 (19.7)	464 (18.8)	-	-
**Present pregnancy**				
Isoimmunization Rh -ve	143 (4.4)	76 (2.7)	-	-
Vaginal bleeding first trimester	445 (13.5)	333 (11.6)	-	-
Date of LMP unknown	208 (6.3)	153 (5.3)	433 (4.7)	385 (4.2)
Nulliparous	973 (29.8)	903 (31.4)	2,739 (29.5)	2,755 (30.3)
Primigravida	491 (14.9)	463 (16.1)	2,891 (30.4)	2,890 (31.8)
Maternal height in cm	157.2 (6.8)	156.6 (6.6)	156.7 (5.5)	156.4 (6.5)
Maternal weight at first visit in kg	61.4 (13.7)	61.1 (13.2)	58.7 (12.1)	58.3 (11.8)
Duration of gestation at first visit, in weeks	15.6 (8.0)	15.7 (7.8)	16.8 (8.6)	16.1 (8.0)
Late booking for antenatal care (>28 weeks)	368 (11.2)	294 (10.2)	1,285 (13.9)	1.052 (11.6)

**Table 2:** Crude and adjusted relative risks of perinatal outcomes

**Outcome**	**New model**	**Standard model**	**Crude relative risk (95% CI)**^**†**^	**Adjusted relative risk (95% CI)‡**
	**N (%)**	**N (%)**		
Perinatal mortality	234/11672 (2.0)	190/11121 (1.7)	1.20 (1.04, 1.38)	1.18 (1.01, 1.37)*
Neonatal mortality	73/11511 (0.6)	71/11002 (0.7)	0.93 (0.80, 1.09)	0.95 (0.81, 1.11)
Fetal Death	161/11672 (1.4)	119/11121 (1.1)	1.30 (1.04, 1.62)	1.27 (1.03, 1.58)*
Fetal death <=36 weeks	122/11574 (1.1)	77/11048 (0.7)	1.63 (1.29, 2.08)	1.64 (1.27, 2.11)*
Fetal death >36 weeks	37/11574 (0.3)	42/11048 (0.4)	0.81 (0.52, 1.26)	0.78 (0.51, 1.21)

* RR estimates using robust variance Poisson modeling, taking clustering into account.

† Model adjusted for risk strata only.

‡ Multivariate model adjusted for risk strata, smoking, education less than primary, hospital admission in last pregnancy, previous surgery on reproductive tract, late booking, vaginal bleeding in the first trimester, age, previous low birth weight, parity.

Table 3 shows adjusted relative risks for fetal, neonatal and perinatal mortality, stratified by maternal risk. Perinatal mortality was not significantly different in high-risk (Adjusted RR 0.94; 95% CI 0.79, 1.12) or low-risk women (Adjusted RR 1.24; 95% CI 0.95, 1.63). Within the high-risk group, the new model was associated with an increase in the overall risk of fetal death (Adjusted RR 1.78; 95% CI 1.33, 2.39) and an increased risk of fetal death at less than 36 weeks (Adjusted RR 1.48; 95% CI 1.17, 1.89). In the low maternal risk group, the new model was associated with an increase in the relative risk of fetal death at less than 36 weeks (Adjusted RR 1.56; 95% CI 1.05, 2.31), however the overall risk of fetal death was not significantly different (Adjusted RR 1.26; 95% CI 0.91, 1.73). The risk of neonatal mortality was not significantly different in low-risk women RR 1.35; 95% CI 0.87 – 2.10) but was significantly lower in high-risk women (Adjusted RR 0.47; 95% CI 0.30, 0.72). We tested baseline maternal risk as an effect modifier, however this was not significant for any model (data not shown).

**Table 3:** Crude and adjusted relative risks of fetal and neonatal outcomes stratified by risk and model of antenatal care

**High maternal risk group (n = 5,125)**				
**End points**	**New model**	**Standard model**	Crude relative risk (95% CI)*^0^	Adjusted relative risk (95% CI)*^†^
	**N (%)**	**N (%)**		
Perinatal mortality	91/2713 (3.35)	71/2412 (2.94)	1.00 (0.85 – 1.17)	0.94 (0.79 – 1.12)
Fetal death	65/2713 (2.40)	36/2412 (1.49)	2.12 (1.53 - 2.94)	1.78 (1.33 – 2.39)
Fetal death with GA <=36 weeks	51/2713 (1.9)	26/2412 (1.1)	1.77 (1.48 – 2.10)	1.48 (1.17 – 1.89)
Neonatal deaths	26/2648 (1.0)	35/2376 (1.5)	0.48 (0.31 – 0.72)	0.47 (0.30 – 0.72)
**Low maternal risk group (n=17,668)**				
**End points**	**New model**	**Standard model**	Crude relative risk (95% CI)*^0^	Adjusted relative risk (95% CI)*^‡^
	**N (%)**	**N (%)**		
Perinatal mortality	143/8959 (1.60)	119/8709 (1.37)	1.14 (0.90, 1.44)	1.24 (0.95, 1.63)
Fetal death	96/8959 (1.07)	83/8709 (0.95)	1.18 (0.91 – 1.52)	1.26 (0.91 – 1.73)
Fetal death with GA <=36 weeks	71/8959 (0.8)	51/8709 (0.6)	1.48 (1.08 – 2.04)	1.56 (1.05 – 2.31)
Neonatal deaths	47/8863 (0.5)	36/8626 (0.4)	1.38 (0.89 – 2.14)	1.35 (0.87 – 2.10)

* Relative risks adjusted for clustering within clinics.

^0^ Model adjusted for risk strata only.

† Model in high-risk women adjusted for risk strata, married or stable union, hospital admission in last pregnancy, previous surgery on reproductive tract, previous low birth weight and vaginal bleeding in the first trimester.

‡ Model in low-risk women adjusted for risk strata, married or stable union, smoking, education less than primary, late booking, age, primigravidity and previous abortion.

**Figure 1:**
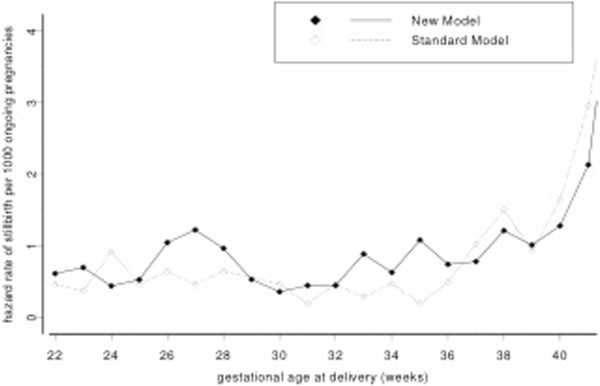
Fetal death hazard rate for gestational weeks 22 – 41.

Stratification of fetal deaths by gestational age (22 – 27, 28 – 31, 32 – 36 and >36 weeks) showed a statistically significant increase in fetal deaths between 32 to 36 weeks of gestation (Adj RR 2.24; 95% CI 1.42, 3.53), the other periods were not significantly different between the comparison groups (Table 4). This pattern is demonstrated in Figure 1, comparing hazard rates for the reduced visits model and standard model.

**Table 4:** Relative risk of fetal death by gestational age

**Gestational age**	**New model**	**Standard model**	**Crude relative risk (95% CI)†**	**P-value**
	**N (%)**	**N (%)**		
22 – 27	52/68 (76.5)	36/54 (66.7)	1.12 (0.98 – 1.29)	0.086
28 – 31	26/77 (33.8)	20/83 (24.1)	1.09 (0.78 – 1.51)	0.62
32 – 36	42/714 (5.9)	20/670 (3.0)	2.24 (1.42 – 3.53)	0.0005
> 36	37/10710 (0.4)	42/10237 (0.4)	0.81 (0.52 – 1.26)	0.35

† Model adjusted for risk strata only.

## Discussion

We conducted a secondary analysis of the WHO ANC Trial dataset following the publication of a Cochrane review that suggested the risk of perinatal mortality was slightly higher in reduced-frequency, goal-oriented antenatal care packages for low-risk pregnancies in developing countries [4]. The analysis found that both high- and low-risk women in the intervention group had an increased relative risk of fetal death between 32 and 36 weeks gestation. While any association between antenatal care interventions and fetal death is cause for concern, these findings are hypothesis-generating rather than conclusive and must be interpreted with caution.

The Cochrane review included data from developed and developing countries and the reviewers acknowledged that there is considerable variation within and between these trials on numbers of visits, making interpretation difficult [4]. While the increase in perinatal mortality in the three cluster-randomized trials was a consistent finding, only the pooled estimate reached borderline statistical significance [4].

Women in these countries differed greatly in baseline health risks and health resource availability, making comparisons problematic. Additionally, the 1996 Zimbabwe trial and WHO ANC Trial were in mostly urban settings [10,13], while the 2007 Zimbabwe trial was rural [14]. This heterogeneity needs to be considered when interpreting the marginal increase in perinatal mortality on pooled analysis. Furthermore, data from the two Zimbabwean trials showed that overall, women had similar numbers of visits between intervention and controls groups, suggesting that any increase in the risk of fetal death in the intervention groups may be due to factors other than the number of antenatal visits [13,14]. Secondary analyses of the two Zimbabwean trials have not yet been published.

In retrospect, the disparities in fetal mortality could have been more thoroughly investigated and given greater prominence in the published findings of the WHO ANC Trial. One possible explanation for these findings is that lower number of visits or other elements of the new antenatal care package decrease the identification of fetuses at risk of fetal death between 32 and 36 weeks. This may be attributable to prolonged gaps between antenatal visits (particularly between the second and third trimester) or issues with the quality of antenatal care delivered. The number of antenatal visits cannot necessarily act as a proxy for high quality care and measuring the effective delivery of antenatal interventions is difficult. Reducing the number of antenatal visits may not necessarily translate into fewer visits of higher quality for more women in low-resource settings. It may also be that control clinics were providing other interventions that were of some benefit.

This analysis has several limitations. Control clinics in the WHO ANC Trial used a regimen based on local protocols; it is therefore likely that visit frequency differed both within and between control clinics. 47.6% of women in the new model and 19.6% of women in the standard model had fewer than five visits, rendering differences in visit frequency negligible for this sub-group. Cluster-randomized trials are an efficient study design for trialing complex interventions in low-resource settings, but it can be difficult to account for all potential confounders that may differ between clusters. For example, antenatal care providers in the WHO ANC Trial were known to differ between study sites and clinics, as well as rates of smoking, education, hospital admission, previous gynecological surgery and late booking for antenatal care [10]. Complete blinding in trials of antenatal care is impossible, increasing the chance of a selection or information bias emerging. However, it is reassuring that after adjustment for identifiable, potential risk factors, the crude and adjusted results are similar. It is also worth noting that in high-risk women the increased fetal mortality was accompanied by a statistically significant drop in neonatal mortality (Adjusted RR 0.47; 95% CI 0.30, 0.72). While the reasons for these findings are unclear, it could be secondary to the higher number of fetal deaths during pregnancy.

In many low-income countries, where resources are limited, antenatal care coverage is poor and many women receive little or no antenatal care [15], and as such focusing on the provision of effective antenatal care at a reduced frequency seems advantageous. On the other hand, additional antenatal visits may serve to reinforce maternal education and compliance, or provide an opportunity for screening and treatment of a condition that had been missed, omitted or deteriorated since the previous visit. The reduced visit model has been adapted and implemented in the north-east region of Thailand and is currently being scaled up to the whole country. The model has been modified with an added visit around 20 weeks for an ultrasound scan. Initial monitoring of perinatal mortality has not revealed any adverse outcomes in the north-east region. (P Lumbiganon, personal communication).

## Conclusion

This secondary analysis of the WHO ANC Trial data indicates there is an appreciable increased risk of fetal death at 32 to 36 weeks gestation for women receiving the goal-oriented, reduced frequency antenatal care package. While it is plausible that this was due to reduced antenatal visits, differences in settings, populations or content and quality of care as well as the timing of visits could also be playing a role; care must be taken in comparing findings across varied settings, countries and patient groups.

It is critical to monitor maternal, fetal and neonatal indicators when implementing antenatal care protocols in any setting. In settings where few women attend antenatal care, achieving four antenatal care visits with the full complement of targeted, evidence-based interventions at each visit is still meaningful. These programs should be monitored not only in terms of number of visits but also in terms of actual care delivered and clinically meaningful maternal and perinatal outcomes. Further research on rates of fetal death in centers using reduced antenatal care packages would also be of benefit.

### Details of ethics approval

The WHO Antenatal Care Trial was approved by the Scientific and Ethical Review Group of the UNDP/UNFPA/WHO/World Bank Special Programme on Research, Development, and Research Training in Human Reproduction, the WHO Secretariat Committee for Research into Human Subjects, and the Institutional Review Boards of the individual participating centres and corresponding health authorities of the regions where the trial was implemented.

## Competing interests

The authors report on competing interest. Several of the authors (GP, GC, PL, HB) were also co-authors in the original WHO Antenatal Care Trial.

## Authors’ contributions

This analysis was conceived of by several authors (JS, AMG, TD, GC, HB, PL, GP, OTO) following the WHO technical consultation in November 2010. NH performed the analysis. The draft manuscript was written by JV. All authors contributed to and approved interpretation of the findings and the final manuscript. The views contained herein are those of the named authors only.
